# Validation of the food access survey tool to assess household food insecurity in rural Bangladesh

**DOI:** 10.1186/s12889-015-2208-1

**Published:** 2015-09-07

**Authors:** Muzi Na, Alden L. Gross, Keith P. West

**Affiliations:** Department of International Health, Johns Hopkins Bloomberg School of Public Health, Center for Human Nutrition, 615 N. Wolfe St., W2041, 21205 Baltimore, MD USA; Department of Epidemiology, Johns Hopkins Bloomberg School of Public Health, Baltimore, Maryland USA

## Abstract

**Background:**

Perception-based Likert scale are commonly used to assess household food insecurity. The aim of this study was to evaluate the psychometric properties and external construct validity of the 9-item Food Access Survey Tool (FAST) in a population-based randomized controlled trial.

**Methods:**

Participating women (*n =* 11,992) were asked to recall the frequencies of nine food insecurity experiences over the past 6 months on a 5-point Likert scale. The Rasch partial credit model was used to study the item category severity and differential item functioning (DIF) by literacy status, respondents’ age, land ownership and household sizes. Principal component analysis (PCA), non-parametric methods, and cumulative ordinal logistic regression models were applied to examine the Rasch model assumptions, namely unidimensionality, monotonicity and measurement invariance (non-DIF).

**Results:**

All items demonstrated good model fit with acceptable values of fit statistics (infit). PCA as well as other indices (Cronbach’s alpha = 0.85, scalability coefficient = 0.48) indicated that all items fit in a single statistical dimension. The ordered responses of nine items displayed monotonic increasing item category severity as expected theoretically. All nine items were flagged with statistically significant DIF between key demographic—and socioeconomic subgroups (*p <* 0.001); however, none of the detected DIF was considered practically significant given small effect sizes (variance explained by group membership and interaction term < 1 %). The total summed score over the polytomous FAST was inversely associated with household wealth, dietary diversity score and maternal body mass index, demonstrating external construct validity.

**Conclusion:**

The polytomous FAST is internally and externally valid tool to measure household food insecurity in rural Bangladesh. Validation of this type of studies are recommended for similar Likert food insecurity scales.

## Background

Food security is defined by the Food and Agriculture Organization of the United Nations (FAO) as “when all people, at all times, have physical, social and economic access to sufficient, safe and nutritious food that meets their dietary needs and food preferences for an active and healthy life” [1]. Food security can be defined at national, regional, household and individual levels [2]. These scales all rely on three concepts in a hierarchical order: food availability, access, and utilization [3]. In the past two decades, researchers have created and tested many perception-and behavior-based Likert scales to capture food access at the household level, which lacks direct measurable indicators [4]. The notion that the coping behaviors reflect severity is rooted from the theory that food insecurity and hunger are “managed processes” [5]. Perceptions and behaviors evolve with progressive difficulty in food access, ranging from psychological concerns to actual compromises in food quality and quantity. The goal of food security scales is to order households along a food insecurity continuum based on how frequently each household displays a range of coping behaviors [2].

The Rasch model, a one-parameter logistic model under item response theory, is recommended [6] and is frequently used in household food insecurity scale development and assessment of the individual items, or questions, that make up the scale [7–12]. The Rasch models estimate food insecurity by allowing different items to have different severities which reflect different stages of household food insecurity. The probability of endorsing an item on a logit scale is modeled as a linear function of the overall household food insecurity score and the item’s severity. Rasch models assume unidimensionality, monotonicity and local independence of the items within a scale. If those model assumptions hold, there is a desirable property of ordering the latent household food insecurity by a simple summed score of affirmed responses. A continuous food insecurity score from a polytomous scale may allow the use of information from all response category. Previous validation studies employing Rasch modeling have demonstrated the internal validity of using the total number of affirmed items of food insecurity scales with dichotomized indicators. However, research gaps exist in understanding the psychometric properties and model assumption fitness of the polytomous items measuring food insecurity.

Therefore in this study, we examined the internal validity and external construct validity of the total score of a polytomous scale against common indicators of food insecurity. The example we use of a Likert food insecurity scale is the Food Access Survey Tool (FAST) designed by the Food and Nutrition Technical Assistance group for use in Bangladesh [13].

## Methods

### Subjects and data collection

For this validation study, we used data from women who had participated in a cluster-randomized trail comparing efficacy of antenatal multiple micronutrient versus iron-folic acid supplementation on a range of birth and postnatal outcomes in Gaibandha District in rural northwestern Bangladesh. Details of the parent trial are described elsewhere [14]. Briefly, newly pregnant women of reproductive age (13–45 years) consented to participate in the trial and demographic and socio-economic status (SES) were collected at enrollment via a standardized questionnaire. Women were then visited at home at 3 months postpartum, asked about the frequency of dietary intake of 32 foods in the previous 7 days, weighed lightly clothed on SECA digital scales (UNICEF) to the nearest 100 g, measured in terms of height using a portable stadiometer and left mid-upper arm circumference (MUAC) with an insertion tape, both to the nearest 0.1 cm. From the data collected at enrollment, a wealth index using selective SES variables was created according to a previous published methodology [15]. Dietary diversity scores and weight status were determined from data collected at the 3 months postpartum follow-up. Woman’s dietary diversity score (WDDS) was calculated following the FAO guidelines [16], ranging from 0 (no food group in the past 7 days) to 10 (maximum diversity). Maternal body mass index (BMI) was calculated as weight/ height^2^ (kg/m^2^). Undernutrition and overweight was defined as a BMI value less than 18.5 kg/m^2^ and more than 25.0 kg/m^2^, respectively.

At 6 months postpartum, the FAST was used to retrospectively assess household food insecurity in the past 6 months [13]. It consisted of 9 items covering several key domains of household food insecurity experiences [4], including: 1) security and predictability over food acquisition (worrying about food, purchasing rice often, and running out of food); 2) reduction in food quality and/or quantity (eating square meals, eating other grains when rice is preferred, skipping meals, eating less food); 3) socially acceptable behaviors or strategies to augment resources in the context of rural Bangladesh (taking food on credit from shops and borrowing food from relatives or neighbours). Participating women were asked to recall the frequencies of the above 9 situations on a 5-point Likert scale: 0 = never; 1 = rarely; 2 = sometimes; 3 = often or 4 = mostly. The question about “square meals” was reversely coded in order to be consistent with higher frequency indicating more severe food insecurity as represented in other items. A total score was created by summing the indexed frequency of all nine items. Households with FAST assessment data entirely missing were excluded, leaving a total sample of 11,992 households included in the analysis.

This study was approved by the national ethical review board, Bangladesh Medical Research Council, Dhaka, and the Institutional Review Board of the Johns Hopkins Bloomberg School of Public Health, Baltimore, Maryland.

### Statistical analysis

We conducted a psychometric validation analysis using the partial credit model, which is an extension of the Rasch model for polytomous responses. The unidimensional partial credit model was chosen because a simple summed score is a sufficient statistic of the latent trait under this model [17]. A simulation study comparing partial credit model with other common polytomous models under the item response theory also suggest small violations of stochastic ordering under various conditions [18]. Therefore, if data upholds model assumptions and fits the partial credit model well, the total summed score should contain sufficient information to order household by the severity of food insecurity. The external construct validity of FAST was examined against common indicators of household food insecurity, such as poverty, maternal food intake and nutritional status.

Specifically, we conducted the following analyses: 1) partial credit model fit assessment; 2) model assumption check; 3) estimation of item category severity; and 4) examination of the FAST external construct validity against proxies of household food insecurity. The R 3.1.1 (The R Foundation for Statistical Computing) was used to conduct all the analyses. The TAM (Test Analysis Modules) package 1.0-3 was applied to build the partial credit models, calculate model fit statistics, and check model assumptions.

#### Model fit of the partial credit model

Under the partial credit model, the probability of a person choosing a response category of an item is modeled as a step logistic model between adjacent categories of both the person’s food insecurity latent and item severity parameter. Two Chi-square type statistics, namely *Infit* and *Outfit*, are used to evaluate the fit of Rasch models. Both statistics compare the estimated responses by the model with the observed responses. *Infit* is a weighted statistic giving more weight to the households’ food insecurity severity around the estimated item severity, whereas *Outfit* is unweighted and is more sensitive to outlying responses. In food security scale analyses, *Infit* is given more priority because it is more informative in population research [9, 12, 19]. The two statistics are commonly expressed as the mean of the summed squared standardized residuals (Mnsq) and standardized t-statistics. Considering stability with sample size for polytomous data, we chose to report squared standardized residuals [20]. A squared standardized residual value of 1.0 is found if the observed data fit the model perfectly. A range of 0.7 to 1.3 is considered acceptable while a narrower range 0.8 to 1.2 is recommended [11]. In this study, we compare *Infit* Mnsq statistics of all item category parameters with both ranges.

#### PCM model assumptions

Belonging to the Rasch model family, there are some key assumptions required: local independence, unidimensionality, monotonicity, and measurement invariance. Local independence states the independent relationship between responses to any two items conditioning on the underlying food insecurity trait. Detecting violations of local independence is challenging [21], therefore this assumption is often assumed or implied from model fit results as did in other validation studies [7, 12, 19]. The examinations of other three assumptions are introduced in more details below.

Unidimensionality refers to a single latent trait being measured by the given scale. In the case of FAST, we expect that only one food insecurity latent trait is necessary to account for the inter-item associations in the data. Various indices have been used to evaluate unidimensionality [22]. Cronbach’s alpha, a measure of internal consistency and the Loevinger’s scalability coefficient (*H*), an indicator of homogeneity, were calculated for the entire scale. A cutoff of 0.7 for Cronbach’s alpha [23] and 0.4 for *H* was used as acceptable level [24]. Principal component analysis (PCA) on polychoric correlation matrix was used to detect the number of components among the FAST items [25]. To help determine the significance of unidimensionality, observed eigenvalues from PCA are compared with simulated eigenvalues from parallel analysis, which is based on item correlation matrix that is due to chance alone. Three criteria were set to identify unidimensionality: a) only one component with observed eigenvalue > 1; b) variance explained by the first component > 2/3 of total variance; and c) the observed eigenvalue > simulated eigenvalue only held true for the first component [22, 26, 27].

Monotonicity indicates that the probability of endorsing a response is a monotonically non-decreasing function of the food insecurity latent trait. It means that households with increasing severity of food insecurity have greater risk of responding to a more severe category than households with less severe food insecurity. The Mokken scale analysis by the R/Mokken package 2.7.5 was used to examine monotonicity. This method compares the probability of endorsing a category or above of the examined item given the sum score of the rest of the items. Detailed methodology and settings are available elsewhere [28]. Briefly, the number of sum score groups is determined by setting the minimum number of subjects per group (denoted as *minsize*) according to sample size. Smaller values of *minsize* results in increased number of sum score groups, which increases the sensitivity of the test as well as the probability of violation detection simply due to sampling error. It is recommended to have *minsize* set at one tenth of the total sample size if sample size is large (>500). In this study where sample size is about 12,000, we applied the recommended level as well as two stricter *minisize* levels at one fiftieth and one over five hundred of the sample size.

Measurement invariance indicates that the response behavior to each item on the scale should not differ by the respondent’s characteristics that are considered exogenous variables of food insecurity, such as respondents’ religion, age, sex, and education level, etc. Measurement invariance of a scale indicates an unbiased scale that is understood similarly across demographic and socio-economic subgroups when they are having similar level of food insecurity. When an item is responded differently by such characteristics, the item is said to have differential item function (DIF). There are two types of DIFs: uniform DIF occurs when an item present consistent degree of DIF across the latent food insecurity trait; however, when non-uniform DIF occurs, the magnitude of DIF varies by food insecurity trait. Because of its ability to simultaneously detect both types of DIFs and its outperformance compared with other commonly used the methods [29], the cumulative logits ordinal logistic regression was chosen to examine DIF in the present study. Detailed procedure is described elsewhere [30]. For each item, proportional odds across all 5 response categories is first tested. Because violations occur in all but one item, we loosened the proportional assumption by applying generalized ordered logistic regression methods. The log odds of response in one category or below is modeled as a linear function of total food insecurity score (model 1) and total score, group membership and interaction term between total score and group membership (model 2). Because model 1 is nested within model 2, log likelihood ratio tests were conducted to test the statistical significance of the total DIFs. The effect size of DIF, defined as the difference of R-squared between model 2 and 1, is used to test the practical significance given large sample size [30]. Significance level is set at p-value of 0.05 and R-squared difference of 13 % for practical significance [30]. DIF was checked across four subgroups, defined by literacy status (literate vs illiterate), age (<30 y vs ≥30 y), land ownership (landless vs land owner) and household size (<4 vs ≥4).

#### Item category severity

Partial credit model was used to estimate the item category severity along the latent trait. In this model, estimated item step severity parameters are the intersection of adjacent category probability curves, which is not immediately interpretable as item category severity. We instead calculated the Thurstonian thresholds, which is the food insecurity latent score on logit scale at which the probability of choosing a category or higher reaches 0.50. The Thurstonian thresholds represent item category severity as its definition is similar to the item severity definition of the dichotomous Rasch model [31]. Higher response categories (indicating greater frequencies) with increasing severity along the food insecurity latent is expected.

#### External validity

Given the lack of gold standard of household food insecurity, the external validity was tested by comparing the total score of the FAST against the distribution of three proximal indicators: 1) wealth index, a proxy of overall household socio-economic status; 2) WDDS, an predictor of women’s dietary quality and nutrient adequacy [32, 33]; and 3) women’s BMI, which reflects nutritional status of the women living in the sampled households. Wealth index and WDDS were divided by quartiles, with the Q1 representing lowest 25 % values and Q4 representing highest 25 % values. BMI values was grouped into undernutrition, normal and overweight by conventional cutoffs. A non-parametric rank test was performed to test any linear trend in the association between total score and the continuous proxy indicators.

## Results

### Sample characteristics and FAST responses

The key characteristics of the households and respondents are listed in Table 1. In this rural population, the mean (SD) household size was 4.2 (2.0) individuals. About 65 % of them were literate and about a third (31.9 %) of the women were undernourished. Those data were comparable with the rural statistics from the Bangladesh Demographic and Health Survey 2011 [34]. Table 2 lists the original questions on the scale and the distribution of categorical responses. Item 1 was reversely coded to represent “no square meals” experiences. Cumulatively, 46.7 % of the respondents answered negatively to all nine food insecurity experiences.

**Table 1:** Characteristics of the study population

Characteristics	N	Mean (SD) or %	
Household			
Size	11,980	4.2	(2.0)
Dependency ratio^a^	11,979	0.6	(0.4)
Land ownership	11,048	52.6	
Women			
Age, y	11,986	22.8	(5.5)
Muslim	11,983	91.4	
Literate	11,981	64.7	
WDDS, per week	11,454	5.2	(1.7)
MUAC, cm	11,552	23.4	(2.2)
BMI, kg/m^2^	11,532	19.6	(2.2)
Undernutrition (BMI < 18.5)	11,532	31.9	

*Abbreviations: WDDS* women’s dietary diversity score; *MUAC* mid-upper arm circumference; *BMI* body mass index

^a^The dependency ratio was calculated as the number of people aged 0–12 and aged over 50 years living in the family divided by the number of people aged 13–49 years

**Table 2:** Item responses to the FAST scale (*N =* 11,992)

Original question		Item description	Responses (%)				
			Never	Rarely	Some-times	Often	Mostly
In the past 6 months, how often did…							
Item 1^a^	you eat three ‘square meals’ (full stomach meals) a day (not a festival day)	Square meals	2.1	1.7	6.2	7.6	82.4
Item 2	you or any of your family have to eat wheat (or another grain) although you wanted to eat rice (not including when you were sick)?	Have to eat other grains	92.7	3.2	3.2	0.7	0.2
Item 3	you yourself skip entire meals due to scarcity of food?	Skip entire meals	89.0	5.8	4.0	0.9	0.3
Item 4	you personally eat less food in a meal due to scarcity of food?	Eat less	77.3	7.0	10.7	2.9	2.1
Item 5	food stored in your home run out and there was no money to buy more that day?	Run out of food	87.4	6.8	4.7	0.9	0.2
Item 6	you worry about where food would come from?	Worry about food	85.0	5.7	6.1	1.7	1.5
Item 7	your family purchase rice?	Purchase rice often	57.9	8.5	8.2	9.5	15.9
Item 8	your family take food (rice, lentils etc.) on credit (or loan) from a local shop?	Take food on credit	84.1	4.6	6.3	2.6	2.4
Item 9	your family have to borrow food from relatives or neighbors to make a meal?	Borrow food	79.4	12.6	6.9	0.9	0.2

^a^Item 1 is reversely coded in analysis and the description for that reversed item 1 is “no square meals”

### PCM model fit assessment

**Fig. 1:**
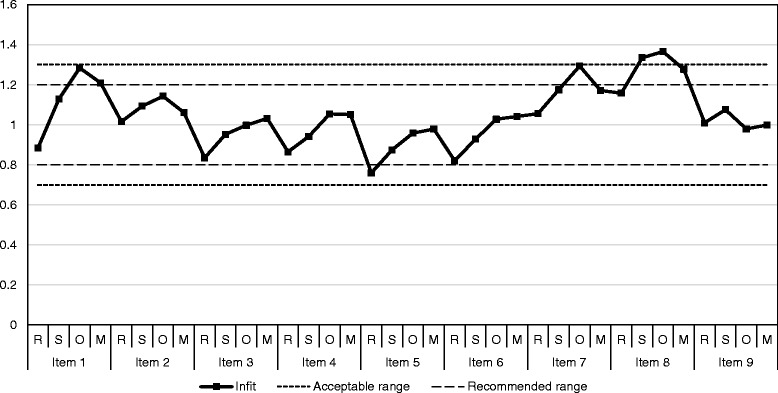
Infit values of FAST polytomous items: R, Rarely; S, Sometimes; O, Often; M, Mostly

*Infit* values after fitting PCM model are plotted in Fig. 1. Among 36 item category parameters, all *infit* values of those parameters were within the acceptable range of 0.7-1.3, except for two of the *infit* values of item 8 “take food on credit” (Sometimes = 1.33 and Ofte*n =* 1.37). If applying the 0.8-1.2 recommended range, there were four additional *infit* values that were out of range: item 1 reversed “no square meals” mostly = 1.21, item 5 “run out of food” rarely = 0.76, item 7 “purchase rice often” ofte*n =* 1.29 and item 8 “take food on credit” mostly = 1.28. In general, *infit* values indicated acceptable fit of polytomous FAST data to PCM.

### PCM assumption assessment

#### Unidimensionality

Cronbach’s alpha for the FAST scale was 0.85 and the scalability coefficient *H* was 0.48. Both values were above the acceptance cutoff and considered strong. The observed eigenvalues from PCA for components were 5.91, 0.85, 0.50, 0.46, 0.40, 0.32, 0.27, 0.15 and 0.15. Variance explained by the first component was 65.7 %. Only the first component had observed eigenvalue greater than the simulated eigenvalues that ranged from 0.96 to 1.04. Results from PCA showed that all the 9 items fit in a primary statistical dimension, which indicated the assumption of unidimensionality was met.

#### Monotonicity

The results from Mokken package are reported in Table 3. The number of active comparison between sum score groups was determined by the number of sum score groups. As the minimum number of subjects per sum score group (*minsize*) decreased, the number of groups went up so did the number of active comparisons. No significant violation of monotonicity was detected if set the *minsize* as at the tenth or the one fiftieth level of the total sample size. When setting the *minsize* at one over five hundred of the sample size, there were 9 to 31 pair comparisons or 1–4 % of total comparisons had local decrease of item response probability as the sum of rest of the items was higher. However, the small proportion of violation was likely due to sampling error because few households have high total score.

**Table 3:** Monotonicity assessment by Mokken with different minimum number of subjects per score group (minsize)^a^

	*Minsize*								
	N/10			N/50			N/500		
	# active comparisons	# violations	# significant violations	# active comparisons	# violations	# significant violations	# active comparisons	# violations	# significant violations
Item 1	40	0	0	312	0	0	1012	18	1
Item 2	40	0	0	344	0	0	938	20	2
Item 3	37	0	0	257	0	0	877	10	1
Item 4	40	0	0	312	0	0	828	7	0
Item 5	21	0	0	228	0	0	730	10	0
Item 6	36	0	0	300	0	0	861	9	0
Item 7	24	0	0	220	0	0	744	17	1
Item 8	40	0	0	312	1	0	1012	24	0
Item 9	36	0	0	268	0	0	865	31	2

^a^N is the total sample size equals to 11992

#### Measurement invariance

The Likelihood ratio tests indicated statistically significant DIF of all items by all four subgroup comparisons, except for item 2 “have to eat other grains” (*p =* 0.05) and item 4 “eat less” (*p =* 0.12) comparing respondents of younger or older age (Table 4). However, effect sizes of all comparisons were less than 1 %, which meant that the additional group membership and the interaction term did significantly improve model fit but did not explain much of the remaining variance. The small effect sizes were all smaller than the cutoff of practical significance.

**Table 4:** Likelihood ratio test between two nested cumulative ordinal logistic regression models for DIF examination

		Literacy (literate vs illiterate)				Age (≥30 y vs <30 y)			
	Model 1^a^	Model 2^b^	−2D	*p*-value	Effect size^c^	Model 2^b^	−2D	*p*-value	Effect size^c^
Item 1	–5515.64	–5496.01	39.27	<0.001	0.18 %	–5508.65	13.98	<0.001	0.05 %
Item 2	–3043.47	–3015.94	55.07	<0.001	0.60 %	–3040.42	6.10	0.05	0.00 %
Item 3	–3359.23	–3348.06	22.34	<0.001	0.16 %	–3348.15	22.16	<0.001	0.20 %
Item 4	–6012.05	–5991.26	41.56	<0.001	0.14 %	–6009.88	4.32	0.12	0.00 %
Item 5	–3244.66	–3220.84	47.64	<0.001	0.33 %	–3240.99	7.34	0.03	0.03 %
Item 6	–4281.38	–4256.09	50.59	<0.001	0.29 %	–4268.56	25.64	<0.001	0.14 %
Item 7	–10278.28	–10184.37	187.82	<0.001	0.57 %	–10245.95	64.66	<0.001	0.18 %
Item 8	–6046.82	–5989.04	115.54	<0.001	0.68 %	–6035.00	23.64	<0.001	0.14 %
Item 9	–6296.15	–6262.50	67.29	<0.001	0.32 %	–6286.59	19.11	<0.001	0.10 %
		Land ownership (Yes vs No)				Household size (≥4 vs <4)			
	Model 1^a^	Model 2^b^	−2D	*p*-value	Effect size^c^	Model 2^b^	−2D	*p*-value	Effect size^c^
Item 1	–5515.64	–5216.83	597.62	<0.001	0.23 %	–5502.33	26.62	<0.001	0.09 %
Item 2	–3043.47	–2904.33	278.28	<0.001	0.30 %	–3031.35	24.24	<0.001	0.22 %
Item 3	–3359.23	–3218.90	280.65	<0.001	–0.22 %	–3349.25	19.95	<0.001	0.14 %
Item 4	–6012.05	–5714.07	595.96	<0.001	0.01 %	–5993.87	36.35	<0.001	0.11 %
Item 5	–3244.66	–3137.21	214.91	<0.001	–0.46 %	–3235.91	17.50	<0.001	0.08 %
Item 6	–4281.38	–4103.63	355.50	<0.001	–0.21 %	–4269.68	23.41	<0.001	0.10 %
Item 7	–10278.28	–9533.13	1490.29	<0.001	0.89 %	–10206.68	143.19	<0.001	0.40 %
Item 8	–6046.82	–5735.46	622.72	<0.001	0.09 %	–6033.31	27.00	<0.001	0.10 %
Item 9	–6296.15	–5888.44	815.41	<0.001	0.17 %	–6282.91	26.48	<0.001	0.04 %

^a^Model 1: The log odds of response in one category or below is modeled as a linear function of total food insecurity score only

^b^Model 2: The log odds of response in one category or below is modeled as a linear function of total food insecurity score, group membership and interaction between total score and group membership

^c^Effect size is defined as the difference of R-squared between model 2 and model 1.c. Effect size is defined as the difference of R-squared between model 2 and model 1

#### Item category severity

**Fig. 2:**
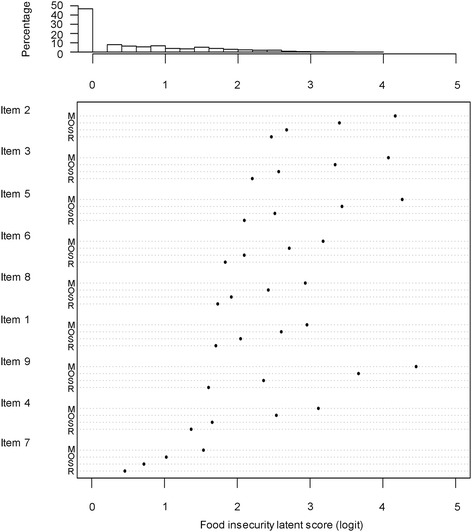
Distribution of estimated household food insecurity latent score and item category severity (Thurstonian thresholds): R, Rarely; S, Sometimes; O, Often; M, Mostly

In Fig. 2, the histogram on the top represents the distribution of households by their food insecurity latent score on a logit scale. The high bar to the far left represented the 46.7 % households that had reported no food insecurity related experiences to the scale. On the bottom of Fig. 2, item category severity (Thurstonian threshold) of FAST items were reordered by the category severity of the “rarely” response. Item severity estimates for the “rarely”, “sometime”, “often”, and “mostly” category were derived from the step-wise PCM and displayed increasing severity from “rarely” to “mostly” for all items. Estimates of category severity were also dispersed along the logit scale of food insecurity latent trait as expected. Logit is the log-odds transformation of the probability of affirming a given category of a scale item. The logit value is expected to be low when the item response category reflects mild food insecurity and high when the category reflects more severe food insecurity. In Fig. 2, the category severity in logit for “rarely” ranged from 0.48 in item 7 “purchase rice often” to 2.49 in item 2 “have to eat other grains”. This means households employed different coping strategies at different level of insecurity: when food insecurity was relatively mild, households reallocate resources to purchase rice more often with smaller amount each time; when food insecurity became worse, household members started to sacrifice their amount of food consumed by eating less (item 4), having no square meals (item 1 reversed), skipping the entire meal (item 3) and even changing their usual grain preference (item 2). Social acceptable strategies in food acquisition (item 9 “borrow food” and item 8 “take food on credit”) were taking place in between the coping strategies of consumption modification. Contrary to the theoretical expectation, however, worrying about food (item 6) did not happen at early stage of food insecurity reflected by a relative higher item severity.

### External validity assessment

**Fig. 3:**
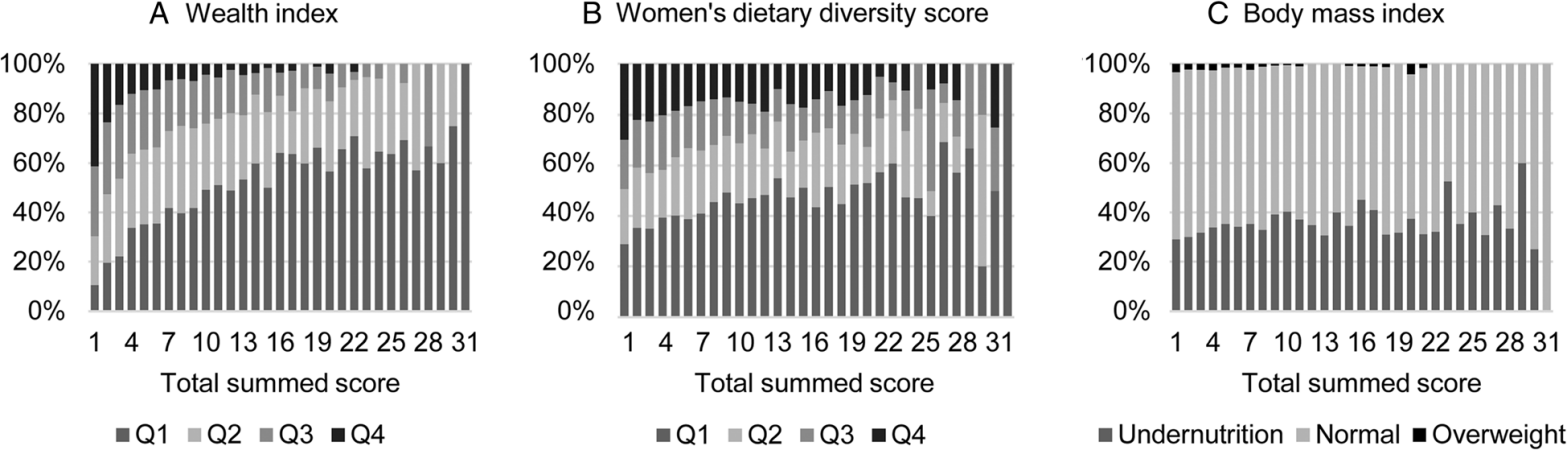
The relationship between the summed food insecurity score and the indicators of household food insecurity: (**a**) wealth index; (**b**) women’s dietary diversity score; and (**c**) women’s body mass index (Q1: 0-25^th^ percentile; Q2: 25−50^th^ percentile; Q3: 50-75^th^ percentile; Q4: 75-100^th^ percentile)

The association between the total score of FAST and wealth index, WDDS and maternal BMI is displayed in Fig. 3. Total score was inversely associated with the three proxies, though dose-response pattern was more apparent in the wealth index histogram. The non-parametric rank test resulted z-scores of −47.9, −19.2 and −9.9 for significant negative linear trend observed in the continuous wealth index, WDDS and women’s BMI, respectively, with increasing total food insecurity scores (all *p <* 0.001).

## Discussion

Using data from a large population-based study, we have demonstrated both internal and external validity of using the sum score of polytomous FAST for assessing the level of household food insecurity severity in rural Bangladesh.

The 9-item FAST was developed by comprehensive qualitative and quantitative research [7, 13]. It shares several common features with other existing perception-based scales in their short-length nature (9–11 items) and their ability to cover multiple key domains representing progressive stages of food insecurity [4]. One uniqueness of the FAST is its inclusion of a social acceptability domain that has items related to resource augmentation strategies under food insecurity. Consistent with previous validity work for the dichotomous items [7], the additional domain fit well with other domains in one primary statistical dimension. And the ordered responses of all 9 items followed the monotonicity assumption. DIF were flagged on the items on FAST as also found in the previous work [7]. Statistical significance was reached because of large sample size but none has reached practical significance. Model fit results indicated a generally good fit of the data to the partial credit model, which also suggested that the assumptions of the extended Rasch model were likely met.

The item severity were toward the expected theoretical sequence with one exception of the item related to worrying about food. It is believed that psychological concerns happens first when the family senses the threats and constraints to food acquiring [5]. In our sample, “worrying about food” seems to happen after a few coping strategies had already taken place, such as eating less, borrowing food, and eating less square meals. One explanation could be our female only sample. In Bangladesh, women are culturally less in favor of intra-household allocation and may sacrifice food quantity when household food insecurity is mild in order to protect her husband and children [35–37]. In addition, due to different social roles by sex in maintaining food security [38], women are more likely than men to borrow food from the kin and neighbors, as demonstrated in the discordance in the item response of female and males under the same households [39]. Because borrowing food is socially acceptable and readily accessible, women may apply such coping strategies at early stage of household food insecurity. On the other hand, if the food insecurity was more chronic than transit in the context, the perception over food acquisition could adapt to high stress situation and remain relatively low in terms of the anxiety level. Given the above reasons, it may be justified that “worrying about food” reflect more severe level of food insecurity in our female sample in rural Bangladesh.

The total summed score of dichotomized items was validated against common food insecurity comparators such as poverty, food consumption, adult BMI and child malnutrition under 12 years old [13]. In our study, we also observed external validity between the total polytomous score and similar food insecurity indicators. The strong association between wealth index and total score was in line with other validation studies comparing a food insecurity index against SES indicators, such as income level [40] and food expenditure [41]. Wealth index was calculated based on a variety of SES indicators capturing a relative static livelihood in this rural setting. The results indicate that food insecurity in rural Bangladesh is a poverty-rooted problem. The weaker association with food quality and maternal nutrition were expected because these are consequences rather than drivers of household food insecurity. More factors play complex roles linking household food access to actual intake (e.g. mechanism of intra-houshold food allocation) and nutritional status (e.g. food and nutrition utilization at individual levels) [42].

Our study has some limitations. First, local independence is assumed rather than tested, although the results from appropriate partial credit model fit implied the assumptions of Rasch model may be met. Second, due to constraints in data collection of the original trial focusing on maternal nutrition and health, external validity was not able to be compared with more proximal drivers or consequences of food insecurity at the household level, such as household income and household food consumption. In this regard, as food manager within the household, but as one whose diet and nutritional status may be particularly sensitive to shifts in home food security, the mother may be viewed as both an optimal and sensitive informant. The three more distal indicators we chose for external validity examination still had expected correlation with the food insecurity index. More investigations are needed to explore the complex mechanism connecting household food insecurity, food intake and nutritional status of household individuals.

## Conclusions

The polytomous FAST is an internally and externally valid tool to measure household food insecurity in rural Bangladesh. The satisfactory model fit and model assumption check of the data to the partial credit model add confidence to use a simple summed score over the polytomous items to order households along their food insecurity latent continuum. Similar procedures examining the internal validity and psychometric property of polytomous items are recommended to other food insecurity scales with different types and number of questions and in other contexts.

## Abbreviations

FAST: Food Access Survey Tool
DIF: Differential item functioning
PCA: Principal component analysis
FAO: Food and Agriculture Organization of the United Nations
SES: Socio-economic status
MUAC: Mid-upper arm circumference
WDDS: Woman’s dietary diversity score
BMI: Maternal body mass index
